# Root Hair Sizer: an algorithm for high throughput recovery of different root hair and root developmental parameters

**DOI:** 10.1186/s13007-019-0483-z

**Published:** 2019-09-04

**Authors:** Marjorie Guichard, Jean-Marc Allain, Michele Wolfe Bianchi, Jean-Marie Frachisse

**Affiliations:** 1 Institute for Integrative Biology of the Cell (I2BC), CEA, CNRS, Université Paris-Sud, Université Paris-Saclay, Sciences Plant Saclay, 91198 Gif sur Yvette Cedex, France; 2 Present Address: Centre for Organismal Studies (COS), Universität Heidelberg, Im Neuenheimer Feld 230, 69120 Heidelberg, Germany; 3 LMS, Ecole Polytechnique, CNRS, Palaiseau, France; 4 Inria, Université Paris-Saclay, Palaiseau, France; 5 Unité de Formation et de Recherche Sciences et Technologie, Université Paris-Est Créteil Val de Marne, 94010 Créteil, France

**Keywords:** Root hair, Image analysis, Cell elongation, Root, Phenotyping, *Medicago truncatula*

## Abstract

**Background:**

The root is an important organ for water and nutrient uptake, and soil anchorage. It is equipped with root hairs (RHs) which are elongated structures increasing the exchange surface with the soil. RHs are also studied as a model for plant cellular development, as they represent a single cell with specific and highly regulated polarized elongation. For these reasons, it is useful to be able to accurately quantify RH length employing standardized procedures. Methods commonly employed rely on manual steps and are therefore time consuming and prone to errors, restricting analysis to a short segment of the root tip. Few partially automated methods have been reported to increase measurement efficiency. However, none of the reported methods allow an accurate and standardized definition of the position along the root for RH length measurement, making data comparison difficult.

**Results:**

We developed an image analysis algorithm that semi-automatically detects RHs and measures their length along the whole differentiation zone of roots. This method, implemented as a simple automated script in ImageJ/*Fiji* software that we termed Root Hair Sizer, slides a rectangular window along a binarized and straightened image of root tips to estimate the maximal RH length in a given measuring interval. This measure is not affected by heavily bent RHs and any bald spots. RH length data along the root are then modelled with a sigmoidal curve, generating several biologically significant parameters such as RH length, positioning of the root differentiation zone and, under certain conditions, RH growth rate.

**Conclusions:**

Image analysis with Root Hair Sizer and subsequent sigmoidal modelling of RH length data provide a simple and efficient way to characterize RH growth in different conditions, equally suitable to small and large scale phenotyping experiments.

## Background

**Fig. 1:**
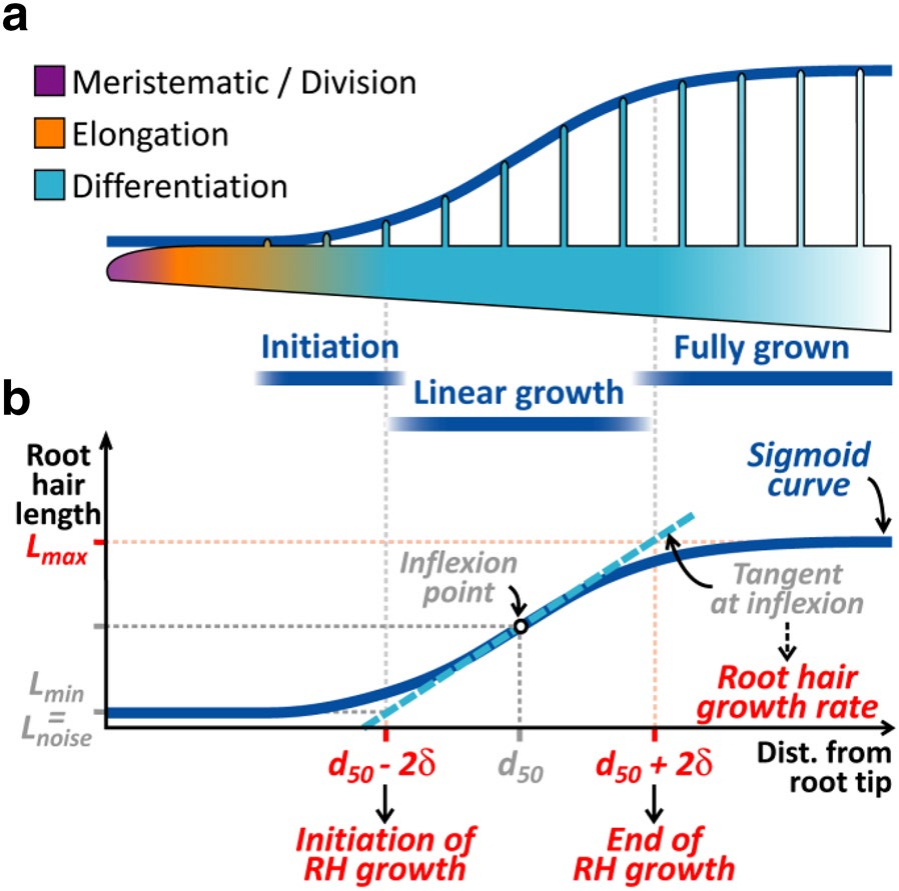
RH development modeled as a sigmoidal curve. **a** Diagram presenting the different developmental zones of the main root (purple, orange and light blue scale) and RHs (dark blue windows). **b** A sigmoidal curve (dark blue line) used to fit the evolution of RH growth. Parameters of RH and root growth extracted from this curve (in red)

Plant biologists employ several macroscopic parameters to describe and study root systems, such as total root length, branching pattern, growth direction and trajectories [1]. Proper analysis of root hairs (RH) is equally important, as these are the main determinants of the root/soil exchange surface, an important site for biotic interactions, and because they constitute an interesting model to study mechanisms governing apical cellular growth [2]. RHs form as single orthogonal cellular extensions of specialized epidermal cells (trichoblasts) as these terminate their elongation, thus their presence defines the differentiation zone of the root (Fig. 1a). In a growing root, division and expansion of cells apical to the differentiation zone propel the apex forward while RHs anchor the root to the substrate. RHs have been studied in order to elucidate several aspects of their development, including general shape description, cytoplasmic architecture, nuclear movement, cytoskeleton and cell wall structure, intracellular trafficking, as well as local reactive oxygen species (ROS) concentration [3–5]. In this work, we focus on RH length measurement; as proposed further, this parameter can be used to deduce some of the aformentioned aspects describing RH and root development.

Several methods describe the measurement of RH length, differing in the approach used to define where to execute the measurement along the root. A first approach simply considers RHs located at a fixed distance from the root tip (RT) [6–9]. However, this method can be erroneous, since the distance of the differentiation zone from the RT can vary with root growth rate. There is therefore the risk of comparing RHs that emerge at different times before or after the application of a given growth condition. A second approach defines an area including RHs described as fully elongated [10–13]. However, the area for RH measurement is selected by the experimenter, which allows un-necessary biases or errors. Finally, both methods generate data from a narrow root segment only, mostly restrained to the fully-grown RH zone. Moreover, both rely on time-consuming manual measurements, which are unsuitable for large-scale analysis.

Two automated image analysis approaches to RH length measurement have been described. Narukawa et al. [14] and Inoue et al. [15] semi-automatically detected RH using image pixel intensity thresholding. However, several steps require the manual selection of binarization thresholds, and only 3.2 mm of root area was used for the analysis. A second method, developed by Vincent et al. [16], allows a partially automated detection of the area filled by RHs, using of a subset of manually analysed images to train an algorithm for automated detection, employing a machine learning approach. Nevertheless, albeit powerful and time efficient, this method does not presently allow measurement of RH length, nor does it allow to accurately define and standardize the position where the measurement takes place along the root.

In the present paper, we propose a method based on the modelling of RH length values along the root with a sigmoid curve, leveraging on a simple, semi-automated image analysis procedure to efficiently measure RH length while keeping track of the distance of each measurement from the root tip. This approach generates accurate and robust measurements of RH length and several other biologically meaningful parameters, including, under certain conditions, an estimation of RH growth rate.

## Results

### Automated RH measurement along the root tip

**Fig. 2:**
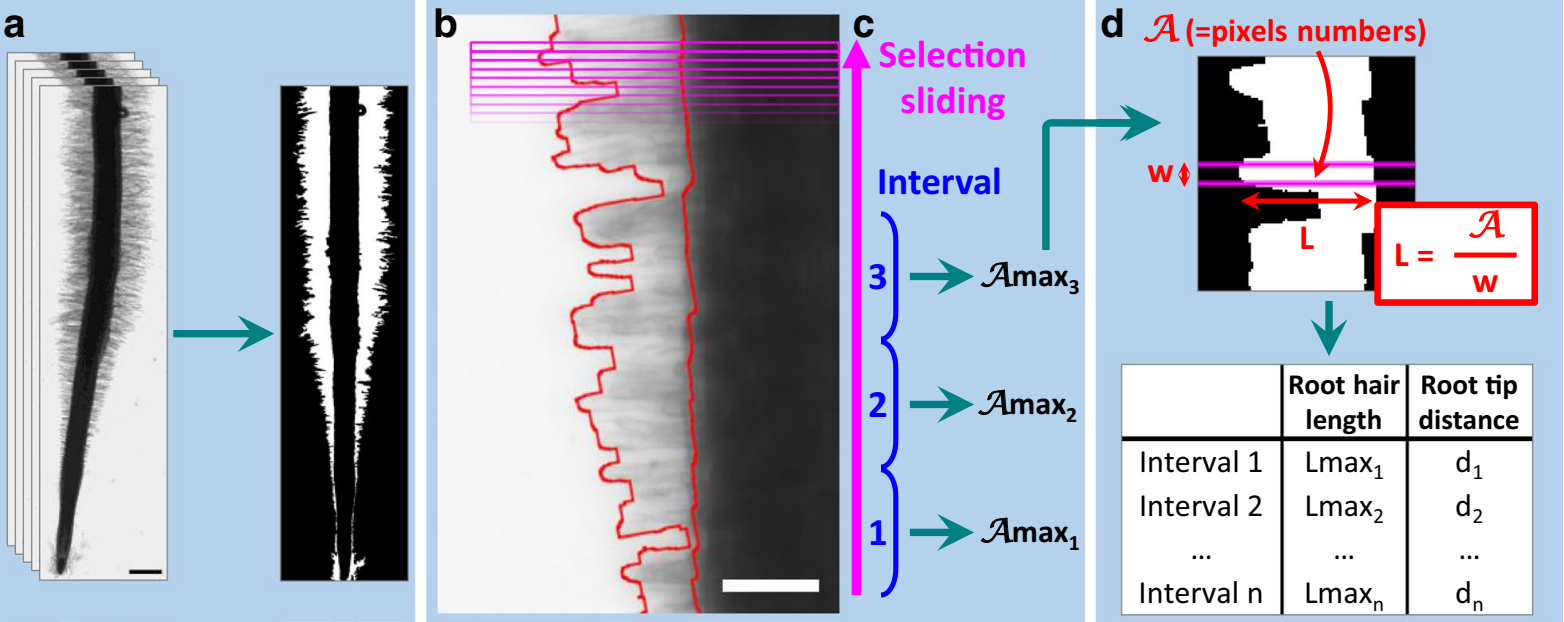
Image processing for root hairs thresholding and measurement. **a** Example of images before and after image processing to detect RH of *M. truncatula* root. Scale: 500 µm. **b** Comparison between automated detection of RHs (red line) and RHs on the original photo. Scale: 100 µm. **c** Image processing to measure and select maximal RH area in consecutive selections intervals. **d** RH length calculation using RH area in one selection. Magenta box corresponds to selection used to deduce RH length

This method was developed to evaluate RH length in in vitro grown *Medicago truncatula* seedlings*. M. truncatula* produces a high amount of RHs, making their individual measurement challenging, in contrast with *for example Arabidopsis*. The procedure requires as input a root tip image where accessible RHs are in focus and well distinct from the root environment in terms of pixel intensity. To achieve this, we summed planes of z-stacks acquired with bright field microscopy (see “Materials and methods”; other image acquisition methods can be used). The modality to produce the images can be kept as presented here, or replaced by any other suitable modality (e.g.: machine learning [16] or plugin “Trainable Weka Segmentation” of ImageJ [17]). It is important to obtain an image of hairs all along the root stem, and to visualize a root outline whereby one can reference the position of hairs from the root tip. Hence, colour images, scanned images and confocal image are worth considering. This root tip image is processed following the procedure summarized in Table 1 to obtain a binarized image of RHs along the root tip, as illustrated in Fig. 2. The outline of the thresholded area obtained follows clearly the RHs profile as shown in Fig. 2a, b. This binarized root image is then straightened to allow an estimation of RH length by sliding a rectangular measuring selection along the root axis (Fig. 2b). The simple formula

1 $$L = \frac{A}{w}$$

is applied, where *L* is the length of the RH profile, computed by dividing *A*, the tresholded surface within the selection, by the width *w* of the selection, which is set to about ½ of RH diameter (Fig. 2d). Since not all RHs are orthogonal to the root surface and straight, and some hairless spots may exist, a max filter is applied to extract only the greatest *L* values over a defined interval of consecutive measurements, reasoning that these *L*_*max*_ values would be an estimate of the length of the best positioned RHs (Fig. 2c). This process therefore generates a population of RH length values associated with their distance from the root tip (Fig. 2d). The image processing steps are implemented as an automated procedure in the Root Hair Sizer (RHS) script for *ImageJ*, available as Additional file 1: Script 1. The procedure is presented in more detail in “Materials and methods” section.

**Table 1:** Steps in Root Hair Sizer (RHS) algorithm

Step	Description	Main tools, plugins or interfaces used in Imagej	Settings used for analysis presented in this paper
0	Requirement: one channel focused .tif images		Sum projection of a Z-stack containing five slices
1^a^	Definition of custom measurement settings for the analysis:	Dialog boxes interface	
	Position along the root axis where to start the analysis		0 µm
	Position along the root axis where to end the analysis		10,000 µm
	Width of the rectangular selection used to measure RH length		8 µm
	Interval for recovery of maximal RH size values		2, 10, 50 or 250
	Facultative step: give a name to the different class of annotations that the user could do manually in step 9.1 (e.g. Artefact, RHs in one side of the root shorter than the other…) Input folder definition for batch image analysis Output folder definition		
Following steps are executed for each .tif image in input folder			
2	Modification of image type to 8-bit		
3	Generation of a binary image of the root with its RHs		
3.1^b^	Thresholding of step 2 image	*Auto Local Threshold*—*Bernsen method,* black background	Radius: 5
3.2	Shape smoothing		
3.2.1^b^	Dilatation and erosion of step 3.1 image	*Dilate* and *Erode*	5 times
3.2.2	Holes filling in image 3.2.1	*Fill Holes*	
3.3	Storage as ROI	*Analyze Particles*—particles size: 0 to infinite pixels and *ROI Manager*	
4	Generation of a binary image for the root body alone (without RHs)		
4.1^b^	Thresholding of step 2 image	*Auto Local Threshold*—*Phansalkar method,* white background	Radius: 100
4.2	Shape smoothing		
4.2.1^b^	Dilatation and erosion of step 4.1 image	*Dilate* and *Erode*	10 times
4.2.2	Holes filling in step 4.2.1 image	*File Holes*	
4.2.3^b^	Erosion and dilatation of step 4.2.2 image	*Erode* and *Dilate*	12 times
4.3	Background cleaning of step 4.2.3 image using the inverted selection of the root (with RHs) defined at step 3.3	*ROI Manager* and *Fill*	
4.4	Inversion of step 4.3 image	*Invert*	
4.5	Storage as ROI	*Analyze Particles*—particles size: 0 to infinite pixels and *ROI Manager*	
5	Generation of a binary image for the area covered by RHs alone (without root body)		
5.1	Subtraction of step 4.4 Image from step 3.2.2 image	*Image Calculator*	
5.2	Background cleaning of step 5.1 image using the inverted selection of the root (with RHs) defined at step 3.3	*ROI Manager* and *Fill*	
5.3	Storage as ROI	*ROI Manager*-*XOR*	
6^a^	Manual suppression of obvious thresholding errors remaining in the step 5.2 image^a^	Dialog box interface and *Brush*	
7	Storage in a ROI of the RH shape resulting from step 6	*Create Selection* and *Make Inverse*	
8	Definition of a root axis line (Root median line, RML)		
8.1	Skeletisation of step 4.4 image	*Skeletonize*	
8.2	Recovery of the skeleton longest path	*Analyse Skeleton* (*2D*/*3D*)	
8.3^a,c^	Convert skeleton step 8.2 image into a segmented line^c^		
	Manual definition of the RT on this line^a^	Dialog box interface	
8.4^b^	Simplification of step 8.3 segmented line		1 segment kept every 200
8.5^a^	RML adjustment	Dialog box interface	
8.6	Storage as ROI of automated and manually curated RMLs	*ROI Manager*	
9	Definition of artefacts or any other comments on image		
9.1^a^	Rough contouring of the zones to be highlighted	*Polygon Selection*	
9.2^a^	Naming (using remarks defined at step 1) of the highlighted zones	Dialog box interface	
9.3	Storage as ROI of rough contours from step 9.1	*ROI Manager*	
9.4	Recovery of minimum and maximum positions of the rough contours along RML analysing the triangle ABC defined by one point of rough contour (apex A), and one segment of the RML (apexes B and C). AI is the median of the triangle (emerging from A), with I the intersection between AI and BC. D is the closest point from A on RML. Steps 9.4.1 to 9.4.3 are done for each point of the rough contour		
9.4.1	Measurement of AI lengths for all RML segments and determination of the shorter one		
9.4.2	Segmentation pixel by pixel of the closer RML segment and measurement of the distance to the apex A of each pixel. The pixel on RML associated with the shorter distance is called D		
9.4.3	Measurement of the distance between the RT and D		
9.4.4	Recovery of minimal and maximal lengths measured at step 9.4.3 for all rough contour points to store them in an array written to the results table		
10^b^	Straightening of image obtain at step 5.2 using the custom RML from step 8.5 as a guide, with line width large enough to cover the root thickness	*Straighten*	RML segmented line width: 1500 pixels
11	Step 10 image binarization	*Make Binary*	
12	Division by 255 of pixel intensities in step 11 image	*Divide* − 255	
13	Step 12 image 90° rotation	*Rotate*	
14	RH measurement. This step is done between the positions defined at steps 1, measuring first the left side of the root, then the right side		
14.1	Creating a selection with width defined at step 1 on one half of the straighten root image (step 10)		
14.2	Sum intensities of pixels included in the selection (this will give the number of RH pixels) and associate the result with the distance of the selection from the RT	*Measure*—*RawIntDens*	
14.3	Repeat steps 14.1 and 14.2 translating the selection by its width to scan the full length of the root		
14.4	Using the interval width defined at step 1, recover the local maximum value within values measured at steps 14.2 and its distance from the RT. Proceeds the same search on next interval until all values measured at step 14.3 are analysed.		
14.5	Convert pixels number recovered at step 14.4 into RH length: the RH pixels area in scanning selection is approximated to a rectangle with known width. The height of this rectangle can be deduced from the rectangle area formula: height = area/width. The length in pixel is then converted to µm		
15	Create a chart summarizing initial settings, maximal RH lengths in the defined interval and associated distances from the RT. A binary code indicates for each measurement if an annotation was done at step 9.4.4 (0 = no annotation, 1 = annotation present)		
16	Save the chart and the ROI generated all along the process		

^a^Manual step

^b^Step that need to be adjusted in the algorithm source code depending of the processed images; c: strategy inspired from ImageJ discussion that won’t be develop here (http://forum.imagej.net/t/measuring-skeletal-length/1262/9)

### Sigmoid modelling of RH length data

**Fig. 3:**
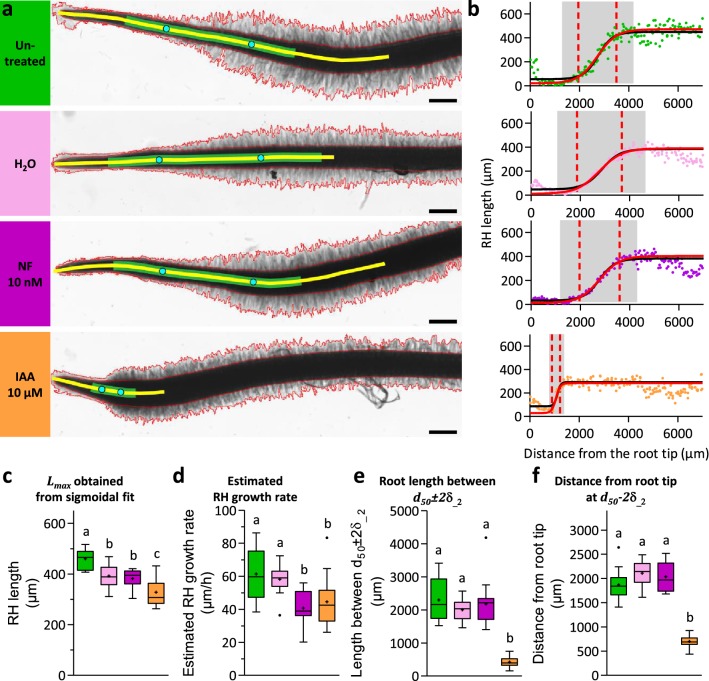
RHS processed on roots submitted to different treatments. Example of application of RHS to analyse the effect of water, NF or IAA treatment on *M. truncatula* roots development. Roots were immersed 1 h in water (pink data), 10 nM NF (magenta data) or 10 µm IAA (orange data) and observed 18 h after immersion. A batch of untreated roots where also observed at the same time (green data). **a** Pictures of representative roots for different tested conditions. Red contours highlight root hairs detected with RHS. Yellow lines indicate root regions considered for the first sigmoidal fit. For untreated and NF treated roots, data obtained between 0 and 6000 µm from the RT were used for the adjustment. For water and IAA treated roots, data from 0 to 5000 µm and from 0 to 2000 µm from the RT were used respectively. Green lines indicate regions used for the second consecutive sigmoidal fit at d_50_1_ ± 5δ__1_. Cyan dots point out d_50_ − 2δ__2_ and d_50_ + 2δ__2_, the initiation and termination of RHs growth. Scale: 500 µm. **b** RH length and sigmoidal curve adjustment of data obtained with pictures presented in **a**. Black and red curves present the two consecutive fits achieved. Grey areas highlight data used to perform the second fit. The first dashed lines mark d_50_ − 2δ__2_ the initiation of RHs growth, the second dashed line mark d_50_ + 2δ__2_ the arrest of RH growth. **c**–**e** Whisker plot comparing, for tested conditions: *L*_*max*_ parameter (**c**), estimated RHs growth rate (**d**), the length between d_50_ ± 2δ__2_ and distance from RT at d_50_ − 2δ__2_ (**f**). Crosses indicate mean value of the corresponding data, dots present outliers according to Tukey method. Data were obtained from two biological replicates, using 7 to 8 *M. truncatula* roots per replicate. For RH growth rate estimation using root growth rate, see “Material and methods”. Letters present the significative groups obtained from a one-way ANOVA test with Bonferroni multiple comparison post-test (p < 0.05)

Once the RH profile is obtained, it becomes possible to extract relevant quantities such as the maximal size of the RHs, the location of the initiation or arrest of the RH growth, etc. To perform this in an automatic fashion, it is convenient to use a simple and realistic law to fit the RHs profile. Our observations of RHs profile show a sigmoid-like pattern (Fig. 3a, b). Indeed, modelling RH length along this region of the root with a sigmoid curve also makes sense in biological terms, when we consider the fact that epidermal cells stop their longitudinal elongation when they start forming RHs. This allows interpreting RH profiles in terms of changes of RH length over time, with an approximately linear phase of RH growth bordered by acceleration and deceleration phases (Fig. 1a, b). For sake of simplicity and robustness we adopted the symmetrical sigmoidal curve *f(d)*

2 $$f\left( d \right) = L_{min} + \frac{{L_{max} - L_{min} }}{{1 + e^{{\left( {d_{50} - d} \right)/\delta }} }}$$

where *d* is the distance from the root tip, *L*_*max*_ and *L*_*min*_ define the higher and lower asymptotes, *d*_*50*_ is the distance corresponding to the mid-point or inflexion point of the curve, and *δ* is a slope factor at inflexion point (Fig. 1b). The value of *L*_*min*_, which would be ideally equal to zero, corresponds to a small amount of noise measured by the image analysis process on the root section devoid of RHs. Hence, for clarity and hereafter, *L*_*min*_ will now be named *L*_*noise*_. This model fit the experimental data presented below with a r^2^ = 0.93 ± 0.03 SD. Several biologically significant parameters can be inferred. Firstly, the length of newly formed, fully grown RHs can be measured as *L*_*max*_. Secondly, the intersections of *L*_*noise*_ and *L*_*max*_ asymptotes with the tangent at the inflection point, placed at d_50_ − 2δ and d_50_ + 2δ, can be used to define in an arbitrary fashion the positions of initiation and arrest of RH growth. Furthermore, these positions also delineate the approximately linear portion of the sigmoid curve, of width 4δ. Since epidermal cells have stopped their elongation in this region, linearity of this central portion indicates that RHs are growing at the same rate. This rate can be easily computed provided that the root growth rate is known (for more details on this, see “Discussion”):

3 $$Growth\; rate_{RH} = \frac{{L_{max} - L_{noise} }}{4 \times \delta } \times Growth \;rate_{root}$$

### Example of application: effect of IAA and Nod factor on RH growth in *Medicago truncatula*

The auxin hormone IAA is a key regulator of root and RH growth [18, 19], while Nod factor (NF) is involved in signalling processes leading to the specific Legume/Rhizobium symbiotic interaction, inducing, among others cellular responses, cytoarchitecture modifications and RH swelling (Additional file 2: Fig. S1, [20]). In order to test Root Hair Sizer, Medicago plants (two biological replicates for each condition, using 7 to 8 *M. truncatula* roots per replicate) were treated either with IAA, NF or water for 1 h, and 18 h later roots were imaged. An untreated batch of plants was referred to as untreated control (Fig. 3a). Images were processed and data points were sampled using an interval of 10 selections as described in “Materials and methods”. In order to fit data with the most accurate sigmoid it was necessary to delimit the set of points to consider. For this purpose, we proceeded in a two step adjustment. The first step was to determine a first rough sigmoid. In order to set the range of positions along the root to take into account for the first sigmoid, we have for each experimental condition, pooled all data points (Additional file 2: Fig. S2). Looking at the data points distribution, we defined a range spanning the root from its very tip up to an arbitrary point within the plateau, corresponding to the maximal length attained by root hairs: 0 µm to 6000 µm for untreated and NF treated roots, 0 µm to 5000 µm for H_2_O treated roots, and 0 µm to 2000 µm for IAA treated roots (Additional file 2: Fig. S2). Following this, the fit of the first sigmoid was performed for each individual root (Fig. 3b, Additional file 2: Figs. S3, S4, black fit) giving a first inflexion point d_50_1_ and an extension length δ__1_. In a second step we computed the definitive sigmoid fit using the range d_50_1_ ± 5δ__1_. Figure 3b, Additional file 2: Figs. S3, S4 exemplify second fits (in red) attesting that we obtained a good estimation of the RH length curve in this region of the root.

Root hair length values defined by the *L*_*max*_ parameter obtained through this two-step sigmoidal fit shows that NF compared with water treatment did not affect RH length. However, the treatment itself, consisting in opening square dishes and incubate root for 1 h in aqueous solution, affects root hairs by reducing its elongation (Fig. 3c). IAA treatment, as expected for a treatment at 10 µM [26], induced a strong reduction in RH length. Provided that the root growth rate is known (statistics and root growth rate provided in “Materials and methods”), the model also offers the possibility to estimate RH growth rate (Eq. 3), a parameter that is usually assessed by time dynamic acquisition on individual hairs. In our growing conditions the RH growth rate of 60 µm/h observed for the control was not modified by water, while IAA and NF treatments slowed down the growth rate to 40 µm/h (Fig. 3d). Another interesting parameter is the length of the RH growth region (d_50_ ± 2δ), which represents the portion of the root along which a RH achieves its full development. RH elongation zones are delimited by the two dashed lines in the sigmoidal fits in Fig. 3b. With a length of about 2000 µm, RH growth regions are equivalent for untreated and H_2_O and NF treated plants, while IAA treatment strongly affects the elongation of this zone which reaches only 500 µm in this condition (Fig. 3e). Finally, as illustrated in the scheme in Fig. 1, we propose the root segment delimited by the root tip and d_50_ − 2δ to be a good approximation of the root division and elongation zones. Therefore, the process of division or elongation (or both) is strongly affected in IAA treated roots compared to the other samples (700 μm versus 2000 μm; Fig. 3f; see [25]).

### Transfer of the method to other species

In order to explore further the possibilities of the algorithm, RHs of two different species were measured. As for Medicago, the monocotyledon *Brachypodium* presents a dense population of root hairs, with a light opaque root corpus (Additional file 2: Fig. S5a). Hence the algorithm required only slight modifications to be adapted to the pictures analysed, mainly by adjusting radius or iteration parameters from the steps 3.1, 3.2, 4.1, 4.2, 8.4 and 10 described in Table 1 (Additional file 3: Script 3, see details of adjustable settings in “Materials and methods” section). The sigmoidal fit applied at the region 0 to 6000 µm from the root tip described well the data obtained with RHS (Additional file 2: Fig. S5a). Alternatively, Arabidopsis roots have a very different profile. The hairs are shorter and less dense and the root corpus is more transparent, leading hence to a different grey level dynamic (Additional file 2: Fig. S5b). Consequently, steps 3 to 5, consisting of the thresholding steps, needed to be modified (Additional file 4: Script 4, see details of adjustable settings in “Materials and methods” section). Additionally, the interval of consecutive measurements used to select *L*_*max*_ was set at 30 instead of 10 in order to adapt the algorithm to the low RH density of such a root. The measured RHs length profile correlates with the image of RHs profile, however the RH length can be locally slightly underestimated when the RH is not growing orthogonally from the root (Additional file 2: Fig. S5b).

## Discussion

### Relevance of the measured parameters

In previous works, the procedure to select the RHs consisted in defining a fixed distance from the root tip or from the first RH [6–9]. Such methods can be inaccurate when comparing roots strongly impacted by a treatment, in which RHs length reaches a plateau at widely different distances from the root tip (as in Fig. 3b). Here we propose a method based on the sigmoidal fit of RH length data along the root axis. For sake of simplicity and robustness we adopted a symmetrical sigmoid to describe the growth profile of RHs. A sigmoidal model gave accurate fits when used with our collection of data generated by our image processing routine Root Hair Sizer (RHS). By applying two consecutive, nested sigmoidal fits in a semi-automatic fashion we were able to obtain four relevant RH parameters: (1) length of mature RHs, (2) distance of emerging RHs from the root tip, (3) length of the RH growth region, and (4) estimated RH growth rate (Fig. 3).

We accurately measure maximal RH lengths *L*_*max*_ and detect experimentally induced changes in length. Hence, this method allows standardizing RH maximal length measured in different physiological conditions, with the robustness deriving from the fit of > 100 data points per root.

In addition, the distance from the root tip to the *d*_*50*_ − *2δ* point allows a definition of the end of the root elongation zone, corresponding approximately to the first emerging RHs [21, 22]. Therefore, the region between the root tip and the *d*_*50*_ − *2δ* point is a convenient and accurate estimate of the cumulated length of the cell division and elongation zones, and changes in its length reflect changes in one or both of these processes. Similarly, the region defined by the *d*_*50*_ − *2δ* to *d*_*50*_+ *2δ* zone reflects the length of the RH growth region.

Root hair growth rate is usually measured using dynamic acquisition of individual RHs elongating between a microscope slide and coverslip. With our method this value can be conveniently estimated from single root tip images. The RH growth rate estimated in this way under our experimental conditions, from 40 to 60 µm/h, is in the same range than the 48 to 84 µm/h measured on individual hairs of *M. truncatula* by Sieberer and Emons [20].

### Large scale screening and application to other plants

Since the Root Hair Sizer method requires only one image per analysed root, it should be suitable to screen a large plant collection. We timed, for the three root images provided (repository link), 84 s per root for the whole processing (from opening the image to obtaining the data point in Excel, with a computer system 1 Processor 4 cores 2.8 GHz, RAM 32Go). This leads to an estimation of 42 roots processed per hour. RHS could also be applied to plant models other than *M. truncatula* with adjustments to parameters mainly at the thresholding steps. Additionally, roots with any RH density can be studied by adapting the sampling interval used to generate RH length data. Another prerequisite is the necessity of imaging RHs growing nearly orthogonally from the root. We successfully used RHS on the monocot *Brachypodium* and on the dicot model plant Arabidopsis to quantify their root hair system.

In the approach presented here, RHs were detected by thresholding pixel intensities. For large scale projects, it could be worthwhile to combine the RHS scanning process and sigmoidal fit of RH length with more flexible methods of RH detection, such as the machine learning based RH detection system developed by Vincent et al. [16].

## Conclusion

Root Hair Sizer is a convenient and efficient method to measure RH length and developmental parameters, based on the semi-automatic analysis of one image per root. Subsequent to image processing and RH detection, sigmoidal fitting of multiple RH length data points along the root allows to accurately and reliably measure changes in RH length, and provides estimates of RH growth rate and epidermal cell elongation/division.

## Materials and methods

### Plant material, culture conditions and root treatments

Wild type *Medicago truncatula*—R108 seedlings, used for this study, were harvested in 2008 at INRA—Montpellier, France, and conserved at − 20 °C in a tube in which empty space was filled with hydrophilic cotton to reduce moisture. Seeds were slightly scarified using sand paper (grit P80) and then surface sterilized 30 min under agitation in a solution prepared dissolving 1/4 of a commercial bleach tablet into 250 mL of water supplemented with 30 µL detergent. Seeds where then thoroughly washed three times in sterile water under a laminar flow. If detergent was still present, additional washing steps were performed. Following the last wash, seeds were kept 1 h in water at room temperature for imbibition. Excess water was removed and seeds were spread randomly on water medium 1% agar (HP 696-*Kalys*). Plates were positioned upside down at 4 °C in the dark for 3 to 4 days of stratification. Finally, germination was achieved at room temperature in the dark overnight, keeping plates upside down to allow a straight root growth outside of agar, and to facilitate further transfer on a new culture medium. Seedlings were then cultivated on modified Fahraeus medium solidified with 1.3% Agar (HP 696-*Kalys*) (modified Fahraeus medium: CaCl_2_ 1 mM, MgSO_4_ 0.5 mM, KH_2_PO_4_ 0.7 mM, Na_2_HPO_4_ 0.8 mM, Fe EDTA 50 μM, 0.1 mg/L of each following microelements: MnSO_4_, CuSO_4_, ZnSO_4_, H_3_BO_3_ et Na_2_MoO_4_, pH adjusted at 7.5 with KOH) [23]. A maximum of 8 seedlings with roots around 1 cm were transferred per square Petri dish (12 cm × 12 cm) containing the culture medium covered by an inert absorbent paper (*Mega International*-USA) to avoid root penetration. During transfer, 125 µL of sterile Milli-Q water was added to each root to prevent dryness before closing boxes with Millipore tape. Black pockets were used to cover the bottom part of the dish in order to protect roots from light. Finally, dishes were positioned with around 45° angle from the vertical in culture rooms maintaining conditions at 24 °C, 60% humidity and 16 h illumination.

To test the RHS algorithm, roots were submitted to different treatments. A first set of untreated plants were cultivated for 3 days after transfer, prior to imaging their roots under a microscope. For treated conditions, 2 days after transfer, dishes were opened and placed horizontally to add 20 mL of treatment solutions composed of either 10 nM Nod factor (NF), or 10 µM Indole-3-acetic acid (IAA) diluted in sterile Milli-Q water. As a control, plants were treated with sterile Milli-Q. After 1 h of root immersion, excess liquid was removed, and dishes were closed and put back in the culture room for 18 h.

*Arabidopsis thaliana* (Col0) seeds were surface sterilized for 3 h with chlorine gas produced by mixing 3 mL of HCl 37% with 50 mL of sodium hypochlorite 10%. Seeds were spread in a square dish (12 cm × 12 cm) filled with 1/2MS medium jellified using 0.8% of Plant agar (*Duchefa*). The dish was stored for 2 days in the dark at 4 °C for stratification and then at 21 °C, 16 h illumination for germination and culture. After 9 days, the seedlings were used for image acquisition.

*Brachypodium distachyon* (Bd21-3) seeds were peeled off from husk using forceps, then sterilized for 15 min on a rocker in 1 mL of sterilization buffer (20% household bleach, 0.1% Tween20) and rinsed 4 times in double-distilled water. The seeds were deposited into a square dish (12 cm × 12 cm) filled with 1/2MS supplemented with 1% agar, with the embryo pole of the long axis of the seed pointing downward. After 2 days of stratification at 4 °C, the dishes were transferred and place vertically into a growth chamber at 28 °C and 16 h of illumination for germination. The seedlings were ready for imaging 3 days after germination.

### Image acquisition

Before describing in detail the different steps of the method, the whole pipeline depicting the entire process that encompass Imaging, *Fiji* Script analysis, data sorting and sigmoid fit is summarized in Additional file 2: Figure S6.

Three days after transfer into the growth chamber (that is to say 18 h after specific treatments if applicable), roots were excised from aerial organs and placed between slide and coverslip in liquid Fahareus medium. A bright field microscope (Leica DMI6000 with motorized stage x, y, z. LAS software, Halogen Leica lamp, 5× Dry objective with 0.15 numerical aperture, Hamamatsu Orca ER-1395 Camera) was used to acquire images with a resolution of 19,703 ppi. RH width is on average covered by 10 pixels. The motorized stage of the microscope and the mosaic reconstitution facilities of LAS software were used to acquire and reconstitute all the analysed images (analogous tools are freely available in *ImageJ* and *Fiji* [24]). The precise selection of the ending of the root zone to acquire is not required, since this selection will be standardized later with the sigmoidal model. To obtain sharp images of RHs along the root, Z-stacks of five images spaced by 100 µm were acquired.

Brightfield images of Arabidopsis roots were obtained with a resolution of 15,631 ppi by placing the opened culture box directly under a Nikon SMZ18 stereoscope, equipped with a 0.5× Nikon SHR Plan Apo WD:71 objective, zoom 8, and Hamamatsu C11440 camera. Three XY positions were acquired per root by manually moving the box under the field of view so that two consecutive images overlapped. No Z-stack was necessary.

Brachypodium seedlings were imaged in between microscope slides and coverslips filled with water, keeping the leaves uncovered from the coverslip. The images were acquired with the same optical system as for Arabidopsis seedlings, with a final magnification of 40×. Two XY positions were acquired per root in order to have 2 consecutive images overlap. No Z-stack was necessary.

XY images obtained for Arabidopsis and Brachypodium were stitched in *Fiji* using the plugin *2D Stitching* [24]. If the image overlap wasn’t sufficient for *2D Stitching* to work properly, the stitching was executed manually using the plugin *MosaicJ* [25].

### Root Hair Sizer: image processing

All image processing was done using *Fiji* open source software, but can be achieved also in *ImageJ* open source software with *AnalyzeSkeleton* and *Auto_Threshold* plugins installed [26–28]. As a starting point, a single image is required where all RHs are focused. Here, we generated Z-projections by summing five acquired slices, but any acquisition and processing method producing a single sharp image of RHs along the root can be employed.

The rest of the processing can be applied by running the RHS algorithm available in Additional file 1: Script 1. A summary of all the algorithm’s steps is also available in Table 1 and illustrated in Figs. 2 and Additional file 2: Fig. S5. The Additional file 5: Movie 1 is additionally available to clarify the understanding of the interface. The first part of RHS processing consists of the detection of RHs area. The method, inspired by Inoue et al. [15], consists of pixel intensity thresholding. A first process will detect the RHs outline, while a second processes the outline of the root only. To avoid manual definition of the pixel intensity threshold, an automated thresholding was used. Detection of root with RHs was achieved using the Bernsen method, whereas the root axis alone was detected by the Phansalkar method (Additional file 2: Fig. S7 and Table 1-steps 3 and 4) [29, 30]. To smooth and fill holes of both binary images generated, several steps of pixel dilatations and erosion were applied (Table 1-steps 3.2 and 4.2). The root axis image was then subtracted from the whole root image (root with RHs) (Additional file 2: Fig. S7 and Table 1-step 5) to obtain the surface covered by RHs alone. Background noise was erased in step 5.2 (Table 1-step 5.2).

As subsequent steps require the root to be straight, the root medium line (RML) is recovered as the longest path of the skeleton obtained from the root axis image after running the ‘*Skeletonize*’ and ‘*Analyse Skeleton* (*2D/3D*)’ commands (Additional file 2: Fig. S7 and Table 1-steps 8.1 and 8.2). The RML image is then converted into a poly-linear selection (steps 8.3 and 8.4), and straightened with *Fiji* ‘*Straighten*’ command (Additional file 2: Fig. S7 and Table 1-step 10). Since ‘*Straighten*’ generates a grey levels image, an additional binarization is implemented (Table 1-steps 11 and 12). In the final image, RH pixels have a value of 1, and background pixels a value of 0.

### Root Hair Sizer: RH length measurement

Root hair length measurement is done in two steps, as detailed in “Results” section (Fig. 2). First, a rectangular selection is slid along the root axis of the straightened, binarized image of RHs, measuring the height “L” of RH surface within the selection at each step (Table 1-steps 14.1 to 14.3) with formula (1). Second, a given number (typically 10) of consecutive individual L values were Max-filtered to obtain a *L*_*max*_ value as an estimate of RH length (Table 1-steps 14.4 to 14.5). Each *L*_*max*_ value is associated with a corresponding distance from the root tip and written to a table.

### Root Hair Sizer: adjustable settings

Depending on the type of root and properties of the acquired image, it may be necessary to adjust some settings. Adjustable steps are highlighted with several asterisks in RHS script, while settings we employed in Medicago examples shown are listed in Table 1. When thresholding the image, the radius value for the Bernsen and Phansalkar thresholding methods can be adapted e.g. for images of different resolution, as well as the number of erosions and dilatations applied directly after. If the root hair profile and the type of optical set up used for the acquisition are very different from the example presented here, the users are encouraged to personalise the thresholding strategy to adapt to their own settings. For Arabidopsis roots for example, the Phansalkar instead of Bernsen thresholding method was used at step 3 to detect RH shape, whereas the Bernsen instead of Phansalkar thresholding method was used at step 4 to detect root shape. Additionally, step 5 was no longer necessary (Additional file 4: Script 4). The root skeletonization process might also need some adjustment. The amount of simplification of the poly-line highlighting the root medium line obtained after ‘*Skeletonize*’ and ‘*Analyse Skeleton* (*2D*/*3D*)’ commands can be adjusted for much larger or smaller images. Finally, in the straightening process, it is important to adjust the width of the poly-line so that it covers all RHs.

When running RHS, a dialog box allows the user to select the root segment to analyse. The width *w* of the scanning selection can be adjusted so that it is slightly thinner than one RH (from 1/2 to 2/3). Finally, the interval size in which *L*_*max*_ should be chosen can also be adapted. An interval of 10 selections to collect *L*_*max*_ seemed accurate in the examples shown here. The effect of varying this parameter, which regulates the amount of data points retained versus data variability due to e.g. “holes” in RH profile, is illustrated in Additional file 2: Fig. S8).

Root Hair Sizer also proposes to annotate the image if some parts of the root need to be removed from the analysis. The position of these comments along the root are recovered in the final table through the annotation of each data point as 1 and 0 (respectively the presence and absence of the comment at this point) on separated columns. Furthermore, at several steps RHS allows the user to check and eventually manually correct the automatic process achieved by the algorithm. All automatic definitions and manual corrections are recorded and saved as region of interest (ROI). After running RHS a first time, it is possible to analyse another region of the root while using ROI associated with the original image by employing the script presented in Additional file 6: Script 2. To run this script, it is important to use the same RML width and to fill the first dialog box with the same comment names used with the main RHS algorithm.

### Data processing

Data collected with RHS are sorted with *Excel* spreadsheet software. For all presented results, regions presenting an artefact (for example, a bubble or dust) were removed from the analysis. For some images, one side of the root presented smaller RH all along the length compare to the other side, mostly for roots growing side by side. In these cases, only the side with longer RH were kept for the analysis. Other specific data sorting or parameters are mentioned in figure legends or in the text. As described above in “Results”, in order to accurately fit data points we proceed with two successive sigmoid fits. Then, as exampled in Figures Fig. 3b, Additional file 2: Figs. S3, S4, we obtained a good estimate of the section of the curve harbouring a sigmoid shape.

Data were fitted with the sigmoidal curve $f\left( d \right)$

4 $$f\left( d \right) = L_{min} + \frac{{L_{max} - L_{min} }}{{1 + e^{{\left( {d_{50} - d} \right)/\delta }} }}$$

This first fit provides the inflexion point d_50_1_ and extension length δ__1_. Then, we settle the limits for the second fit between d_50_1_ ± 5δ__1_.

Sigmoidal adjustment was achieved using *GraphPad Prism* software. Data obtained from each image were treated separately. A least squares fitting has been applied using the following rules to define the fit’s initial values for each considered image:

$$initial\;L_{noise} = L_{absolute\;min}$$

$$initial\;L_{max} = L_{absolute\;max}$$

$$initial\;d_{50} = d at \left( {L_{absolute\;max} /2} \right)$$

$$initial\;\delta = d at \left( {0.1 \times L_{absolute\;max} /2} \right)$$

with *L*_*absolute min*_ and *L*_*absolute max*_ respectively the minimal and maximal RH length measured in all data obtained along the whole root of the considered image.

### Root growth rate measurement

This measurement was done on separate batches of roots in the same condition than for RH length measurement. After 1 h of root immersion in the treatment solution, boxes were emptied, then closed, and the RT position was marked on the box cap with a marker. After 18 h of incubation, the new RT position was marked and the box was scanned. The distance between two consecutive points was then measured using *Fiji* software. The root growth rate can be measured as the ratio of this distance by the time between the two measures. Root growth was measured from two biological replicates giving the following values (mean ± SE): untreated: 296 ± 17 µm/h (n = 26); H_2_O: 304 ± 16 µm/h (n = 26); NF: 225 ± 15 µm/h (n = 23); IAA: 55 ± 3 µm/h (n = 19).

### Statistics

*GraphPad Prism* software was used to achieve statistical analysis and sigmoidal fitting. All details about sample size, biological replicates or leaded tests are specified in the figure legends.

## Supplementary information

**Additional file 1: Script 1.** RHS main algorithm than can be run directly in *Fiji* or *ImageJ*.

**Additional file 2: Figure S1.** Effect of NF on RH shape. **Figure S2.** Data collected with RHS used for a first sigmoidal fit. **Figure S3**. Examples of roots analyzed for non-treated and water immersed conditions as in Fig. 3a and b. **Figure S4.** Examples of roots analyzed for NF-treated and IAA-treated conditions as in Fig 3c and d. **Figure S5.** Algorithm tested on Brachypodium and Arabidopsis thaliana roots. **Figure S6.** Pipeline used to recover different root hair and root developmental parameters. **Figure S7.** Detailed image processing for root hair thresholding. **Figure S8.** Comparison of automated measurements depending on width of interval of selection.

**Additional file 3: Script 3.** RHS main algorithm adapted for *Brachypodium* roots.

**Additional file 4: Script 4.** RHS main algorithm adapted for *Arabidopsis thaliana* roots.

**Additional file 5: Movie 1.** demonstration on the use of Root Hair Sizer algorithm in ImageJ on the three root images

**Additional file 6: Script 2.** RHS sub-algorithm that can be run directly in *Fiji* or *ImageJ* using ROI generated by RHS main algorithm.

## Data Availability

The datasets used and/or analysed during the current study are available from the corresponding authors on reasonable request.

## Abbreviations

IAA: Indole-3-acetic acid (auxin)
NF: Nod factor
RH: root hair
RHS: Root Hair Sizer
RML: root medium line
RT: root tip
