# Supplementary material for: Root Hair Sizer: an algorithm for high throughput recovery of different root hair and root developmental parameters
Source: Plant Methods. 2019 Sep 4;15:104. doi: 10.1186/s13007-019-0483-z (PMC6724272; doi:10.1186/s13007-019-0483-z)
Supplement: Supplementary file 2 — Additional file 2: Figure S1. Effect of NF on RH shape. Figure S2. Data collected with RHS used for a first sigmoidal fit. Figure S3. Examples of roots analyzed for non-treated and water immersed conditions as in Fig. 3a and b. Figure S4. Examples of roots analyzed for NF-treated and IAA-treated conditions as in Fig 3c and d. Figure S5. Algorithm tested on Brachypodium and Arabidopsis thaliana roots. Figure S6. Pipeline used to recover different root hair and root developmental parameters. Figure S7. Detailed image processing for root hair thresholding. Figure S8. Comparison of automated measurements depending on width of interval of selection. [file 13007_2019_483_MOESM2_ESM.pdf]

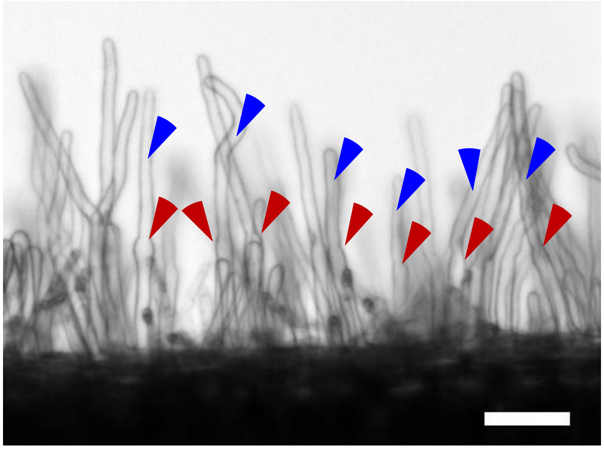

Figure S1. Effect of NF on RH shape

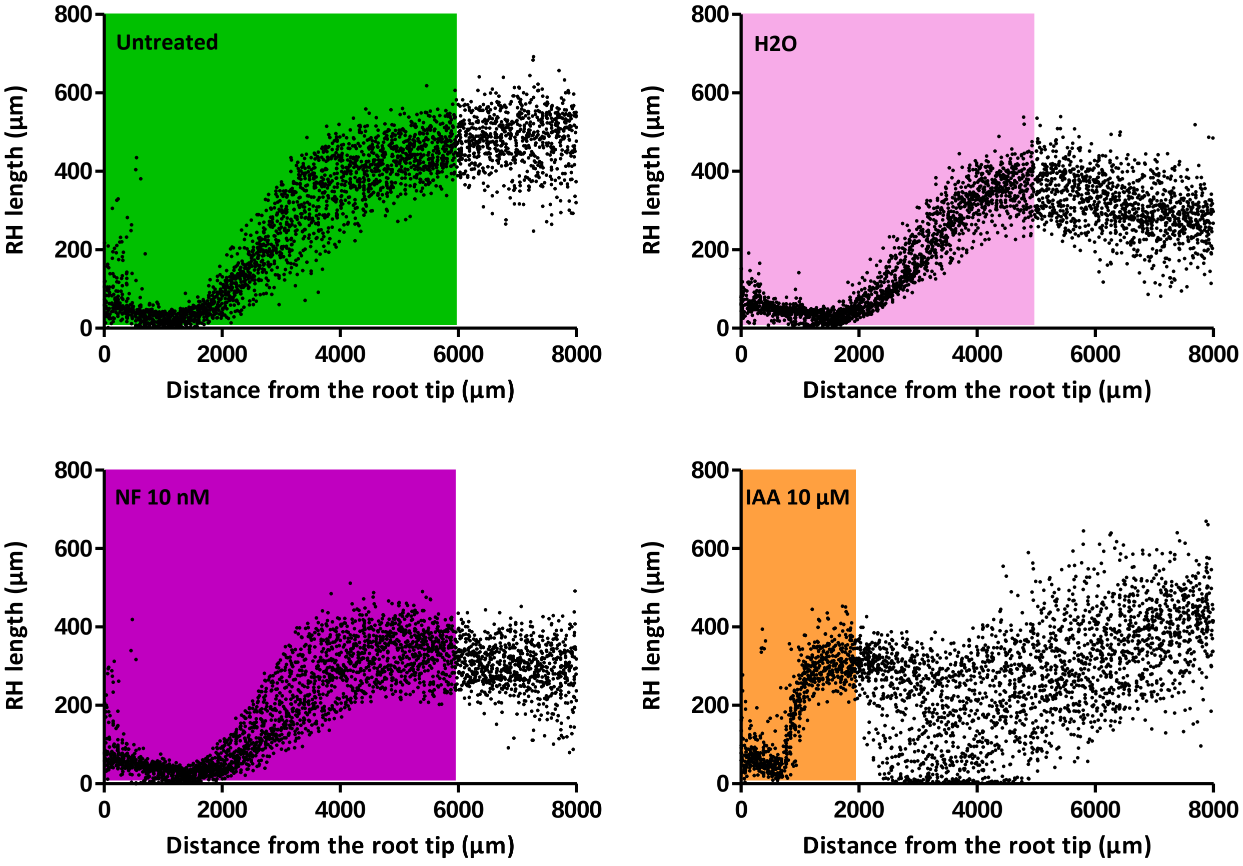

Figure S2. Data collected with RHS used for a first sigmoidal fit

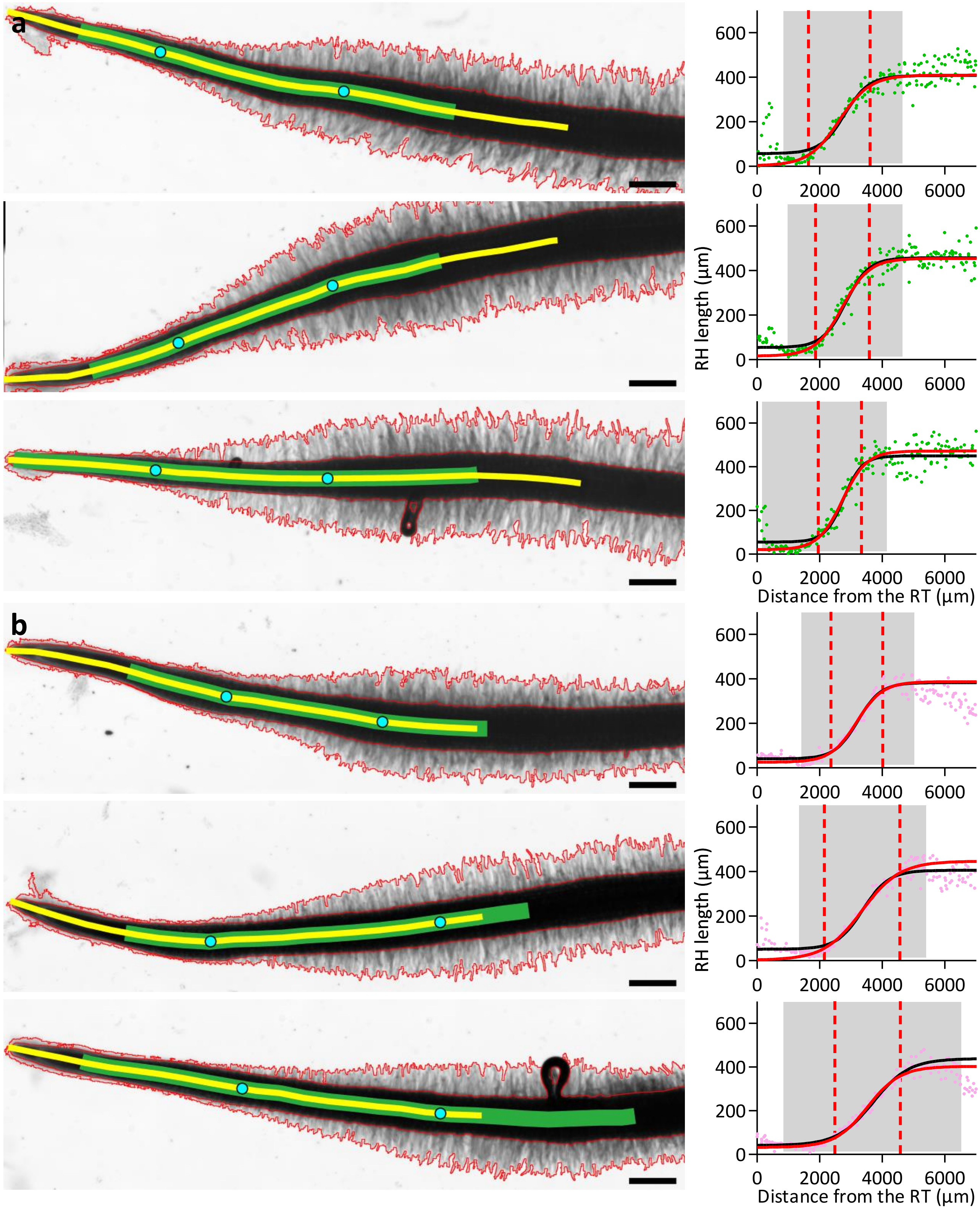

Figure S3. Examples of roots analyzed for non-treated and water immersed conditions as in figure 3a and b

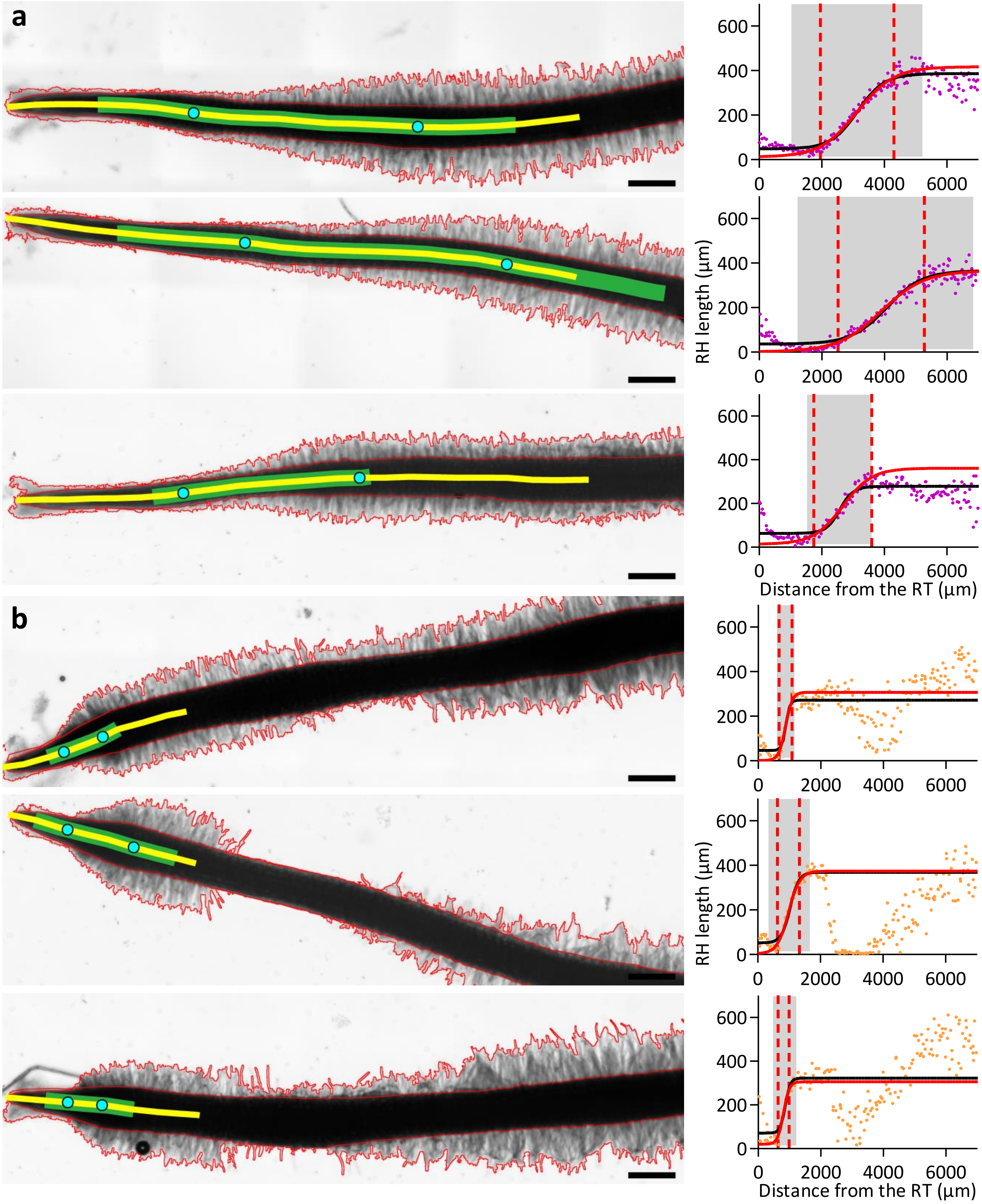

Figure S4. Examples of roots analyzed for NF-treated and IAA-treated conditions as in figure 3c and d

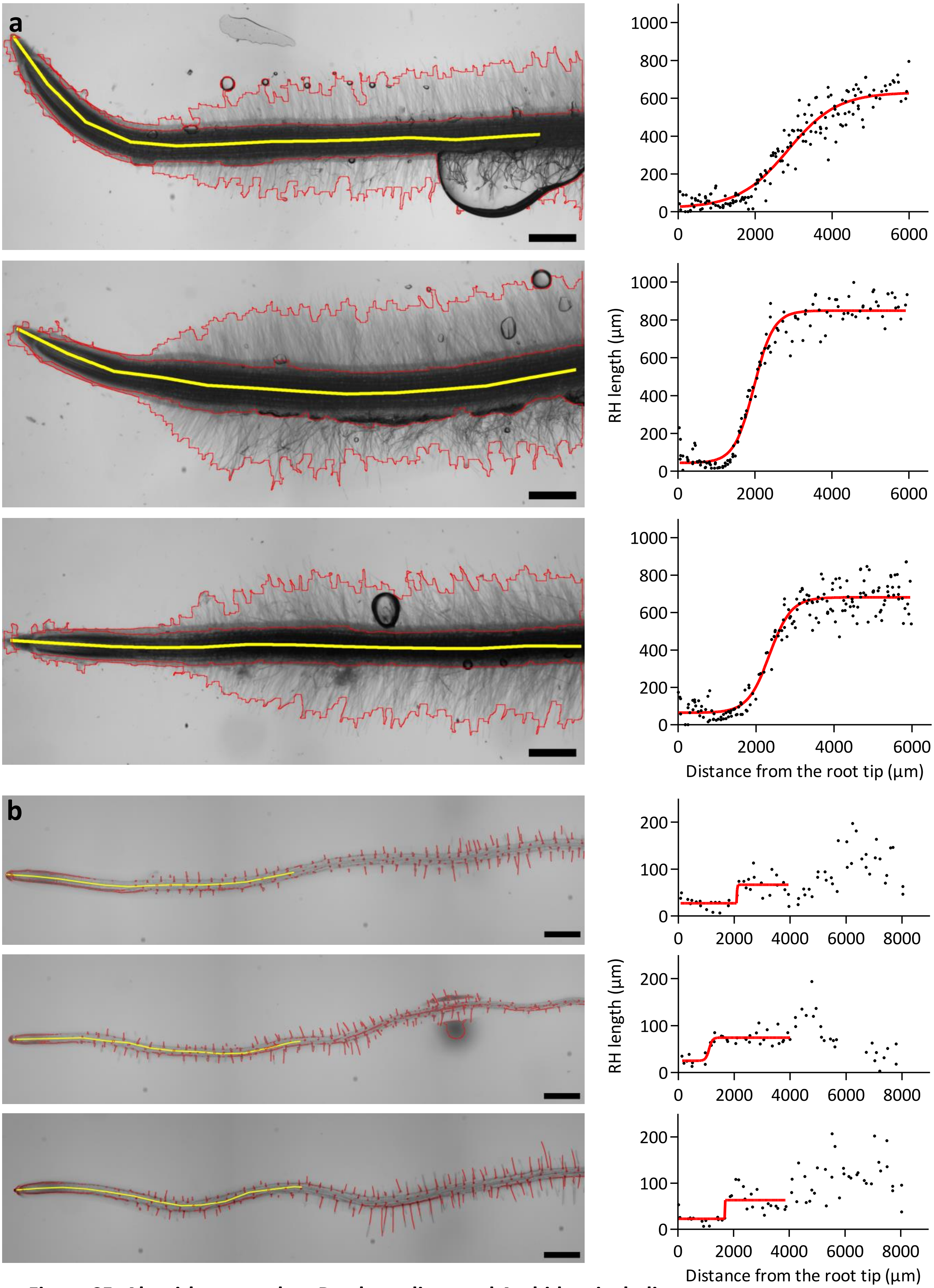

Figure S5. Algorithm tested on Brachypodium and Arabidopsis thaliana roots

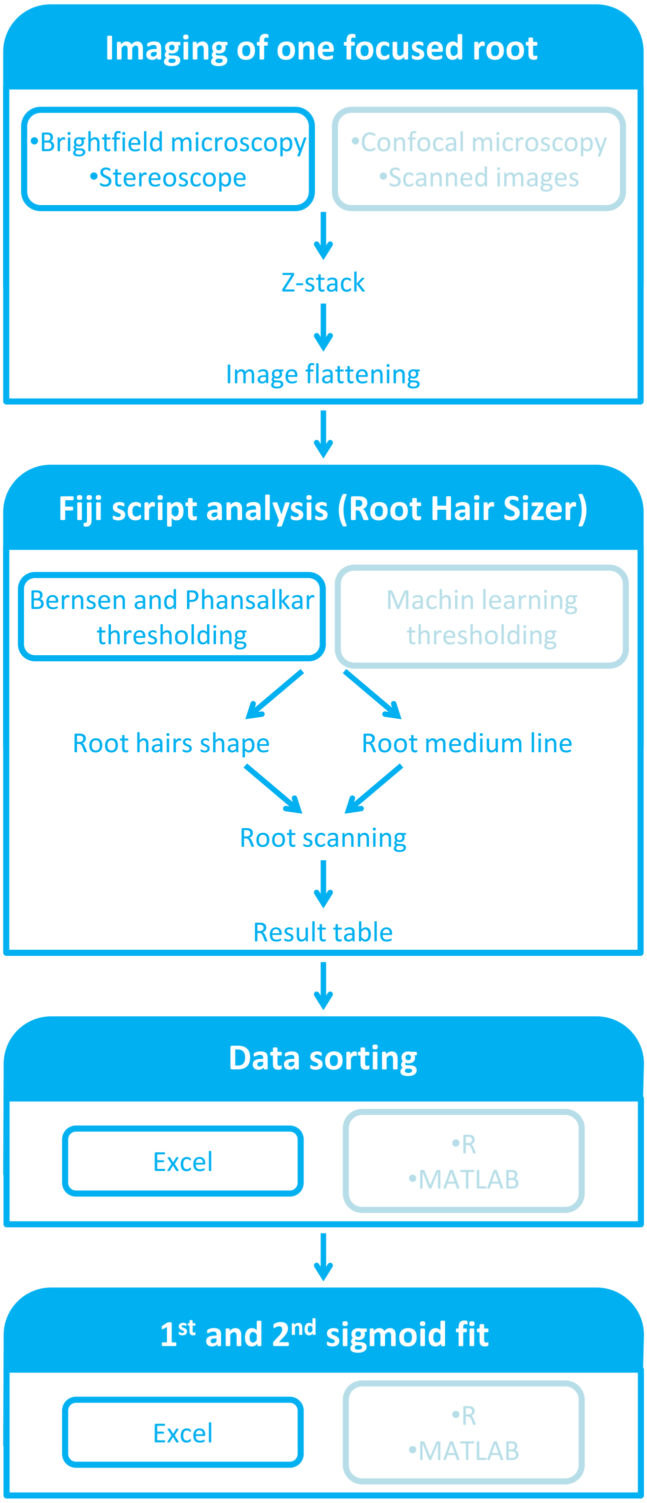

Figure S6. Pipeline used to recover different root hair and root developmental parameters

The pipeline used in this article is present in dark colour. Suggested alternatives are presented in light colour (non-exhaustive list).

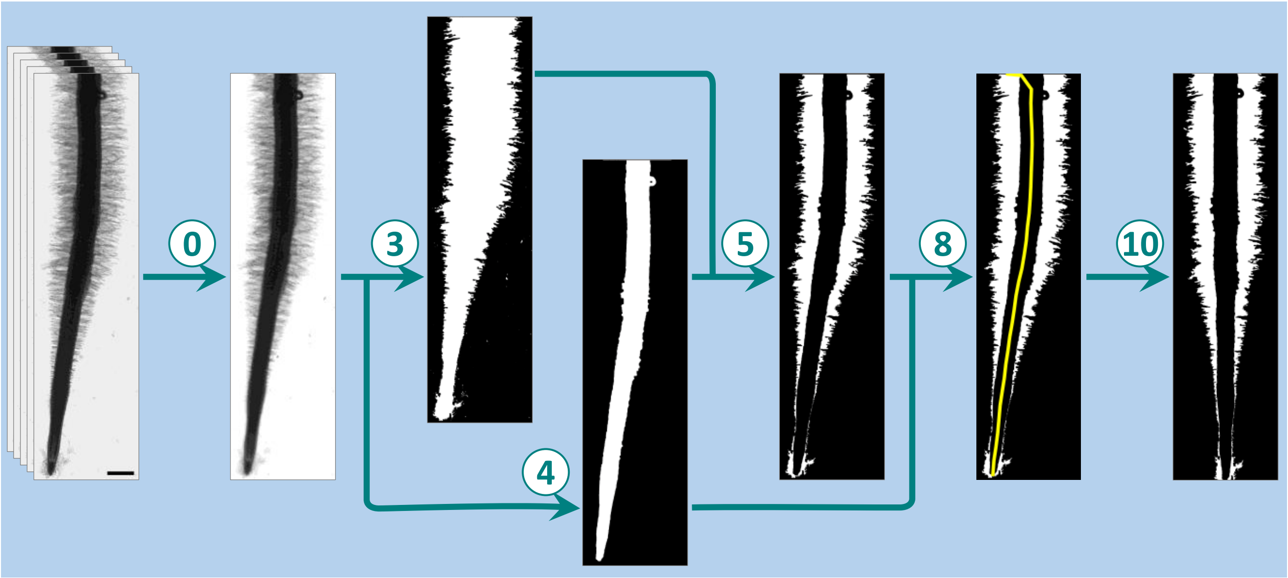

Figure S7. Detailed image processing for root hairs thresholding

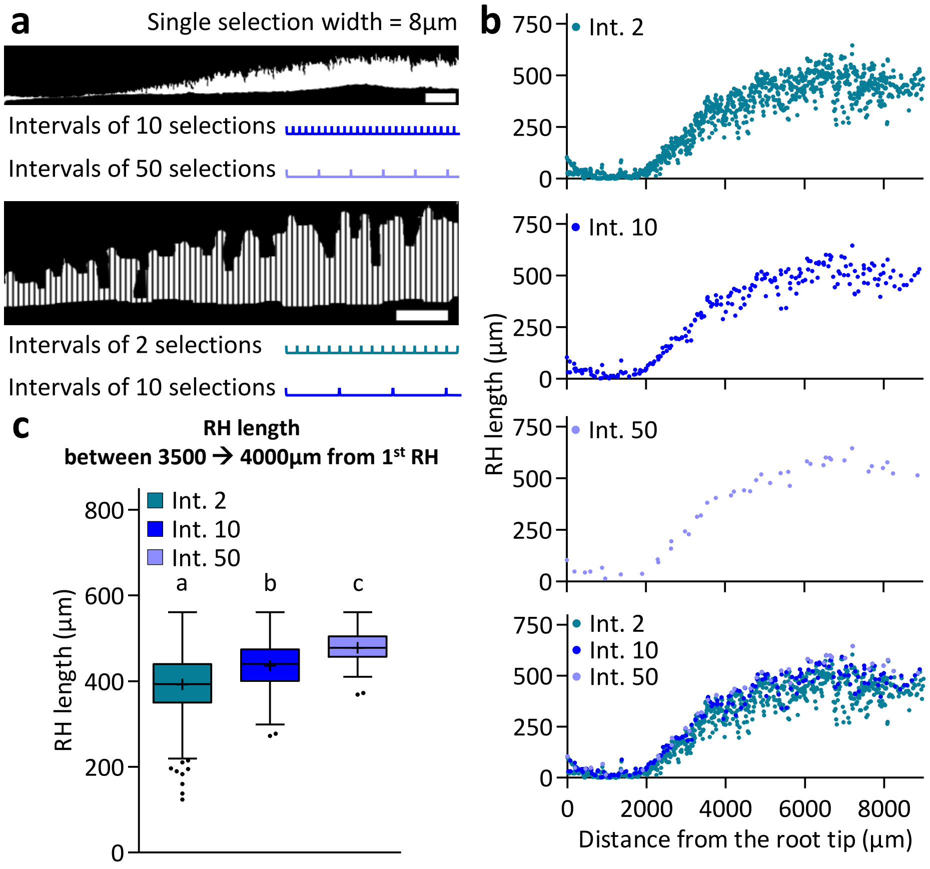

Figure S8. Comparison of automated measurements depending on width of interval of selection
